# Single-Nucleus RNA-Seq Reveals Spermatogonial Stem Cell Developmental Pattern in Shaziling Pigs

**DOI:** 10.3390/biom14060607

**Published:** 2024-05-21

**Authors:** Xiangwei Tang, Chujie Chen, Saina Yan, Anqi Yang, Yanhong Deng, Bin Chen, Jingjing Gu

**Affiliations:** 1 Hunan Provincial Key Laboratory for Genetic Improvement of Domestic Animal, College of Animal Science and Technology, Hunan Agricultural University, Changsha 410128, China; txw@stu.hunau.edu.cn (X.T.); chencj1995@stu.hunan.edu.cn (C.C.); yanganqi90@usc.edu.cn (A.Y.); dengyanhong@stu.hunan.edu.cn (Y.D.); 2 College of Animal Science and Technology, China Agricultural University, Beijing 100193, China; yansaina@cau.edu.cn; 3 School of Basic Medical Sciences, Hengyang Medical School, University of South China, Hengyang 421001, China

**Keywords:** spermatogenesis, single-cell transcriptomics, spermatogonial stem cells, testis, pig

## Abstract

Normal testicular development ensures the process of spermatogenesis, which is a complex biological process. The sustained high productivity of spermatogenesis throughout life is predominantly attributable to the constant proliferation and differentiation of spermatogonial stem cells (SSCs). The self-renewal and differentiation processes of SSCs are strictly regulated by the SSC niche. Therefore, understanding the developmental pattern of SSCs is crucial for spermatogenesis. The Shaziling pig is a medium-sized indigenous pig breed originating from central China. It is renowned for its superior meat quality and early male sexual maturity. The spermatogenic ability of the boars is of great economic importance to the pig industry. To investigate testicular development, particularly the pattern of SSC development in Shaziling pigs, we used single-cell transcriptomics to identify gene expression patterns in 82,027 individual cells from nine Shaziling pig testes at three key postnatal developmental stages. We generated an unbiased cell developmental atlas of Shaziling pig testicular tissues. We elucidated the complex processes involved in the development of SSCs within their niche in the Shaziling pig. Specifically, we identified potential marker genes and cellular signaling pathways that regulate SSC self-renewal and maintenance. Additionally, we proposed potential novel marker genes for SSCs that could be used for SSC isolation and sorting in Shaziling pigs. Furthermore, by immunofluorescence staining of testicular tissues of different developmental ages using marker proteins (UCHL1 and KIT), the developmental pattern of the spermatogonia of Shaziling pigs was intensively studied. Our research enhances the comprehension of the development of SSCs and provides a valuable reference for breeding Shaziling pigs.

## 1. Introduction

Normal testicular development ensures spermatogenesis and produces androgens to maintain male characteristics [1]. Spermatogenesis is a complicated biological process characterized by the mitotic division of diploid spermatogonial stem cells, the meiosis of spermatocytes, and the generation of spermatozoa within the seminiferous tubules [2]. The sustained high productivity of spermatogenesis throughout life is predominately attributable to the constant differentiation and proliferation of spermatogonial stem cells (SSCs) [3]. SSCs, which reside in the basal lamina of the seminiferous tubules, are the most primitive type of undifferentiated spermatogonia, and their self-renewal and differentiation processes are strictly regulated by the SSC niche [4]. The SSC niche, also called the microenvironment, predominantly comprises Sertoli cells, Leydig cells, peritubular myeloid cells, smooth muscle cells, endothelial cells, and immune cells within the mammalian testis [5].

Understanding the developmental pattern of SSCs is crucial for spermatogenesis. However, the high complexity of testicular tissues, which consist of different germ and somatic cell populations, hampers the elucidation of SSC development. The emergence of high-throughput single-cell RNA sequencing (scRNA-Seq) and single-nucleus RNA-Seq (snRNA-Seq) analyses has introduced a new era in male reproductive research [6,7]. Bypassing the intricate nature of testicular tissues, these techniques facilitate the detection of uncommon and heterogeneous cell populations at the single-cell level [8,9]. 

To date, single-cell transcriptome data from thousands of human and mouse testis samples have been analyzed. These samples cover all stages of testicular development from prenatal to postnatal stages, as well as many types of male infertility. These studies have deepened our understanding of the developmental trajectories of SSCs in their niche in human and mouse models [6,9,10]. Presently, the application of single-cell transcriptomics has enabled the investigations of the gene expression dynamics that occur during the development of male germ cells in farm animals, such as chickens [11], goats [12,13,14], sheep [15,16], hybrid cattle-yaks [17,18], yaks [19], and pigs [20,21]. 

The Shaziling pig is a medium-sized indigenous pig breed that originates from central China and is renowned for its superior meat quality and early male sexual maturity, with boars beginning puberty at roughly 75 days and achieving sexual maturity at around 150 days [22]. The fertility and spermatogenic ability of boars are of significant economic significance to the pig industry [23]. Therefore, an in-depth understanding of the process of spermatogenesis and, in particular, the developmental pattern of SSCs in pigs is crucial for pork production, pig breeding, and breed conservation. Prior studies that conducted scRNA-Seq on Guanzhong black pig testicular tissue samples discovered the dynamic processes associated with porcine spermatogenesis and suggested two new cell markers of undifferentiated spermatogonia [20,21]. However, due to differences in genetic backgrounds, the functional development of the testes at the transcriptomic and histologic levels differs significantly between different pig breeds [24]. 

To demonstrate testicular development, especially for the SSC developmental pattern in Shaziling pigs, we utilized single-cell transcriptomics to comprehensively identify gene expression patterns in 82,027 individual cells from nine Shaziling pig testes at three key postnatal testicular developmental stages (neonatal, puberty, and maturity), with three biological replicates at each stage. We obtained an unbiased cell developmental atlas of testicular tissues from Shaziling pigs. We have elucidated the intricate dynamic processes underlying the developmental pattern of SSCs within their niche in the Shaziling pig, thereby offering a valuable reference for Shaziling pig breeding.

## 2. Materials and Methods

### 2.1. Sampling Information

Nine half-sibling Shaziling boars were used to perform snRNA-Seq in this study and testes were collected by castration at three different time points after birth: 1 day, 75 days, and 150 days. We also collected testes from another 24 Shaziling pigs at eight different ages ranging from neonatal to mature, i.e., 1, 10, 20, 40, 60, 75, 110, and 150 days. Testicular samples for each age group were taken from three individuals from the same litter. All pigs were reared in the same farm environment and were provided with standard feeds, with no limitations on their access to water. The collected testicular tissues were cut into small pieces, snap-frozen in liquid nitrogen, and then stored at −80 °C until use.

### 2.2. Histological Analysis and Immunofluorescence Staining of Testicular Tissue Sections

Paraffin-embedded testicular tissues were sectioned into 5-μm thick slides and then stained with hematoxylin–eosin (HE) according to the method described in the previous study [24], which was used to observe the testicular cell development in Shaziling pigs at different ages (in days). Immunofluorescence staining was performed according to our previous study [22] using mouse anti-UCHL1 (1:200; ab8189, Abcam, Shanghai, China) and rabbit anti-KIT (1:100; 3074s, Cell Signaling Technology, Shanghai, China).

### 2.3. Conducting snRNA-Seq

The single-cell nuclei of the testicular tissues were isolated and purified according to Habib et al. [25] with some modifications. In brief, the frozen tissue was minced in 2 mL of ice-cold lysis buffer (Nuclei EZ Lysis buffer, cat #NUC-101, Sigma-Aldrich, Shanghai, China) and incubated for 2 min. Then, the minced tissue was homogenized using a Dounce tissue grinder pestle and incubated for another 5 min with an additional 2 mL of lysis buffer. The homogenates were then incubated on ice for 5 min in RNase-free PBS containing 4% BSA. Samples were then filtered through 100-μm strainers, centrifuged, and resuspended in RNase-free PBS containing 2% BSA and 0.1% RNase inhibitor (cat #3335399001, Sigma-Aldrich, Shanghai, China). Then, each sample was filtered again through 20-μm strainers. Nuclei were counted in the prepared suspension, and the concentration of nuclei was adjusted to 700 to 1200 nuclei per μL. The nuclei, together with barcode gel beads, were then loaded into the 10× Chromium System following the manufacturer’s protocol using the Chromium Single Cell 3′ Reagent Kit v3, 10× Genomics, Shanghai, China. After the completion of library construction, the libraries were subjected to sequencing using the Illumina NovaSeq 6000 platform (Illumina, San Diego, CA, USA) on a 150 bp paired-end mode. 

### 2.4. Data Mapping, Quality Control, and Cell Clustering Analysis

Raw sequencing data from all the samples were processed using the Cell Ranger workflow (version 3.1.0) and mapped to the pig reference genome (Sus scrofa 11.1) by creating a customized “pre-mRNA” reference package (version 3.1.0). The Cell Ranger outputs were then loaded into the R (version 3.5.2) package Seurat (version 3.1.1). Nine datasets were merged, and the quality control filter parameters were set to only retain cells with over 500 expressed genes and less than 25% of reads mapped to the mitochondrial genome. The R package DoubletFinder (https://github.com/chris-mcginnis-ucsf/DoubletFinder, accessed on 1 April 2024) [26] was utilized to remove the doublets from the dataset. UMAPs and cell clustering analyses were conducted on the merged dataset with a resolution set to 0.8. The “FindAllMarkers” function was employed to generate marker genes for each cell cluster. Cell types were then further characterized using proven cell marker genes by examining highly expressed genes specific to each cell cluster. The “DoHeatmap” function was utilized to generate heatmaps of gene expression, which illustrated the highest average log fold change for each cluster corresponding to the top marker genes. To predict the cell cycle phase of each annotated cell type, the “CellCycleScoring” function from the Seurat package was used. The cell types of interest, such as all germ cells and spermatogonia, were re-clustered separately using the “subset” function to generate new Seurat objects. These objects were subsequently subjected to the standard Seurat preprocessing procedure.

### 2.5. Functional Enrichment and GSVA Pathway Analysis

DAVID tools [27] were used for carrying out GO biological process enrichment analysis utilizing a marker gene list specific to each cell type. The msigdbr software package (version 7.5.1) [28] was used to conduct GSVA analysis of hallmark pathways in spermatogonial subsets.

### 2.6. Cell Trajectory Analysis

Pseudotime analysis was performed using the Monocle2 package [29] (version 2.8.0) using the DDRTree method. Based on the pseudotime analysis, branch expression analysis modeling (BEAM) [30] was applied for the analysis of branch fate-determined genes. RNA velocity analysis was performed using Velocyto (version 0.17) [31] for cell fate prediction and estimation of transcription kinetics.

## 3. Results

### 3.1. Overview of Single-Cell Transcriptomes and Histomorphological Analysis of Shaziling Pig Testes at Different Ages 

**Figure 1:**
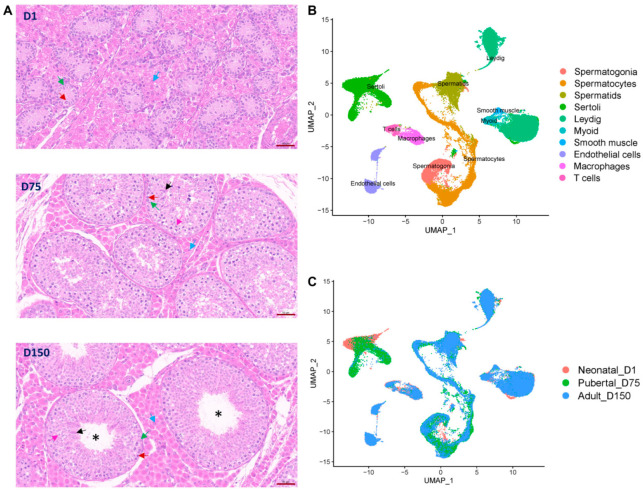
Histomorphological and cell cluster analyses of Shaziling pig testes at different ages. (**A**) Histological examination of testicular tissue sections of D1, D75, and D150. Blue arrow, Leydig cells; green arrow, Sertoli cells; red arrow, spermatogonia; pink arrow, spermatocytes; black arrow, spermatids; *, lumen of seminiferous tubules; and bar = 50 μm. (**B**) The annotated 10 testicular cell types are shown in different colors, including 3 germ cell types (spermatogonia, spermatocytes, and spermatids), 5 somatic cell types (Sertoli, Leydig, myoid, smooth muscle, and endothelial cells), and 2 immunocyte cell types (macrophages and T cells) in the testes of Shaziling pigs. (**C**) UMAP clustering analysis of all testicular cells, with cells colored according to age.

To investigate the development of seminiferous tubules and different types of cells in the testes of Shaziling pigs, sections of testicular tissues were obtained from three distinct developmental stages: neonatal (1 day old: D1), puberty (75 days old: D75), and maturity (150 days old: D150). As shown in Figure 1A and Figure S1, no lumen of seminiferous tubules was observed in D1 samples and only spermatogonia and Sertoli cells were visible within the seminiferous tubules. The existence of a lumen was evident in D75 and D150, with D150 exhibiting a significant enlargement of the lumen. Spermatogonia underwent proliferation and differentiation into spermatocytes and spermatids in D75 and D150. In D150, the haploid flagellated spermatozoa were visible. 

We then performed single-nucleus RNA sequencing (snRNA-Seq) on the 10× Chromium platforms using 82,027 testicular cells from D1 (23,169 cells for neonatal stage), D75 (26,628 cells for puberty stage), and D150 (32,374 cells for maturity stage), each with three biology replicates. We obtained a total of 3145.9 Mb reads from the sequencing libraries. After stringent quality control, high-quality data of 74,578 cells were retained for subsequent analysis. For each cell, the mean reads were 39,584, and the median genes detected were 1918. The Q30 bases in barcodes, RNA reads, and unique molecular identifiers (UMIs) were 95.72%, 94%, and 94.84%, respectively. About 92.32% of reads were mapped to the pig reference genome (Sscrofa11.1), achieving an average of 58.09% sequencing saturation. More information can be found in Supplementary Table S1. 

### 3.2. Identification of Testicular Cell Types by Cell Cluster Analysis 

We distinguished cell clusters via the uniform manifold approximation and projection (UMAP) method and identified each cell cluster using universal mammalian cell type-specific marker genes. We defined 23 potential major cell clusters from D1, D75, and D150, including 8 germ cell clusters, 13 somatic cell clusters, and 2 immunocyte cell clusters (Figure 1B, Figures S2 and S3). The cells in clusters 9 and 13 solely expressed spermatogonial marker genes (*UTF1*, *UCHL1*, and *GFRA1*). Cluster 5, 8, 10, and 15 consisted of cells expressing a range of spermatocyte marker genes (*SYCP1*, *SYCP3*, *RAD51AP2*, *PIWIL1*, *SPATA16*, and *NME8*). The cells in clusters 4 and 14 were expressed spermatid marker genes (*PRM1*, *TNP1*, *TNP2*, and *TPPP2*). As shown in Figure 1C, germ cells in neonatal_D1 samples contained only spermatogonia. Sertoli cells consisted of clusters 1, 6, 7, 16, 18, and 23 and expressed *AMH*, *SOX9*, and *WT1* cell type-specific marker genes. Clusters 2, 3, and 22 were identified as Leydig cells as cells in these clusters specifically expressed *IGF1*, *LHCGR*, *HSD17B3*, *CYP11A1*, *HSD3B*, and *SRD5A1*. Cluster 19 expressed myoid cell marker genes (*MYH11* and *ACTA2*). Cluster 21 expressed a smooth muscle cell marker gene (*NOTCH3*). Cluster 12 and 17 were defined as endothelial cells, expressing *VWF* and *PECAM1* marker genes. We also identified two types of immune cells: cluster 11 expressed the macrophage marker genes (*CD163* and *CD14*), and cluster 20 expressed the T cell marker genes (*CD38* and *IL7R*). The expression patterns of marker genes for the major cell types characterized were also visualized in UMAP plots (Supplementary Figure S4). We successfully annotated 10 testicular cell types in the testes of Shaziling pigs.

To identify the functional categories associated with each annotated cell type, we performed Gene Ontology (GO) biological process enrichment analysis (Supplementary Figure S5). Our functional enrichment analysis revealed that spermatogonia were significantly enriched in the DNA metabolic process, DNA repair and replication, chromatin remodeling, and the regulation of cell development. Spermatocytes were enriched in the spermatogenesis process, gamete generation, and the meiotic cell cycle. Spermatids were involved in peptide and ATP metabolic processes, oxidative phosphorylation, and male gamete generation. Sertoli cells exhibited functions in extracellular matrix organization, ATP synthesis, and cell junction organization. The functional categories of Leydig cells were mainly focused on steroid and cholesterol metabolic processes. Endothelial cells were shown to be involved in cell adhesion and endothelium development. Myoid cells were enriched in epithelial and tissue development. Smooth muscle cells were enriched in mesenchymal development and cell adhesion. Macrophages and T cells were all involved in immune responses. Our functional analyses revealed the functions of various cell types, indicating the reliability of cell type annotation in Shaziling pig testes. 

### 3.3. Discovery of Marker Genes for Each Cell Type in Shaziling Pig Testes 

**Figure 2:**
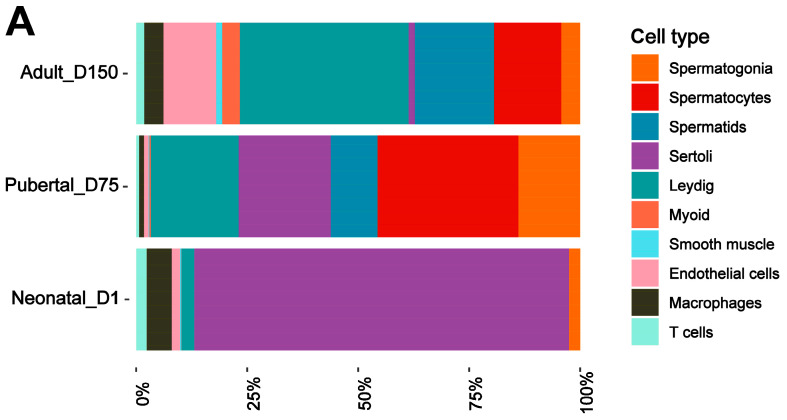

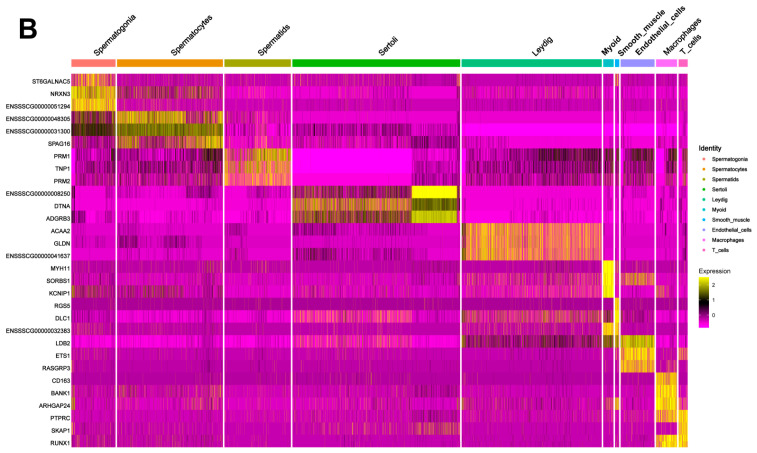
Identification of cell type-specific marker genes in the testes of Shaziling pigs. (**A**) The relative proportions of single cells are classified as different major cell types in the analyzed samples. (**B**) Heatmap of the top 3 marker genes for each cell type. Different cell types are marked by the different colors at the top of the heat map. The left side of the heatmap displays the gene IDs. Color changes from yellow to purple on the right side of the heatmap indicate changes in gene expression levels from high to low.

To gain insight into the development of testicular cells, we counted the relative abundance of each cell type in the three developmental stages of the Shaziling pig testis samples (Figure 2A, Supplementary Table S2). For germ cells, neonatal_D1 samples only contained spermatogonia (2.91%). The proportion of spermatocytes increased from pubertal_D75 (31.67%) to adult_D150 samples (15.29%), while D150 samples had a higher proportion of spermatids (17.96%) than those in D75 samples (10.68%). The number of Sertoli cells declined during testicular development, from 84.22% in D1 samples to 20% in D75 samples, and eventually, down to 1.46% in D150 samples. The proportion of Leydig cells in the D1 samples was low, representing only 3.28% of the total number of testicular cells. This proportion increased to 19.66% in the D75 samples, while in the D150 samples, the percentage increased to 38.11% of the total number of testicular cells. In the D150 sample, the proportion of endothelial cells, peritubular myoid cells, and smooth muscle cells in the surrounding interstitial space was significantly increased, reflecting the characteristics of the testicular microenvironment in mature testicular tissues. A certain percentage of immune cells (macrophages and T cells) were presented in all three stages of testicular development, with the highest percentage in the D1 sample (8.11%), the lowest in the D75 sample (2.1%), and the D150 sample containing 6.25% of immune cells. 

We then generated the gene expression profiles of each cell type. The expression patterns of the top three marker genes were displayed in a heat map (Figure 2B). We identified 1402, 1167, and 424 genes as potential cell type marker genes for spermatogonia, spermatocytes, and spermatids, respectively. In somatic cells, we found 1254 potential marker genes for Sertoli cells, 783 for Leydig cells, 554 for myoid cells, 462 for smooth muscle cells, and 744 for endothelial cells. We also identified 703 marker genes for macrophages and 337 for T cells. These cellular marker genes exhibited specific or high expression levels in corresponding cell types, consisting of a range of known marker genes as *PRM1*, *TNP1*, *PRM2*, *MYH11*, and *CD163*, as well as many potential novel marker genes that provided the ability to differentiate between cell types in the testis as *NRXN3* (spermatogonia), *SPAG16* (spermatocytes), *ADGRB3* (Sertoli cells), and *ACAA2* and *GLDN* (Leydig cells). Detailed information on marker genes is listed in Supplementary Table S3.

### 3.4. Germ Cell Lineage Development in Shaziling Pig Testes 

**Figure 3:**
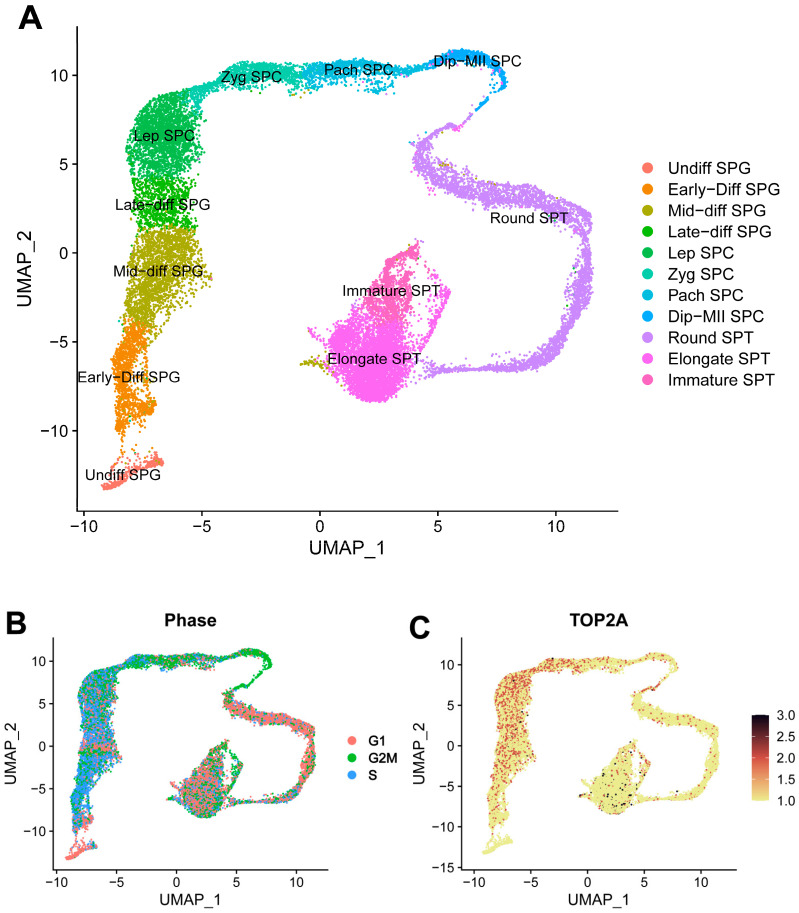
Germ cell lineage development in the testes of the Shaziling pig. (**A**) The 11 different developmental stages of germ cells. Undifferentiated spermatogonia (Undiff SPG), early-differentiating spermatogonia (Early-Diff SPG), mid-differentiating spermatogonia (Mid-diff SPG), late-differentiating spermatogonia (Late-diff SPG), leptotene spermatocytes (Lep SPC), zygotene spermatocytes (Zyg SPCs), pachytene spermatocytes (Pach SPCs), diplotene-to-metaphase II spermatocytes (Dip-MII SPCs), round spermatids (Round SPTs), elongate spermatids (Elongate SPTs), and immature sperms (Immature SPTs). (**B**) Cell-cycle phase analysis of germ cells. The different phases of the cell cycle, including G1, G2M, and S, are represented by dots of different colors. (**C**) *TOP2A* expression demonstrates germ cell proliferation. Changes in *TOP2A* gene expression levels from high to low are indicated by a transition in color from dark red to light yellow on the right side of the UMAP.

To further understand the development of germ cells in the testes of Shaziling pigs, germ cells were extracted and re-clustered. Based on the following marker genes, the progression of germ cell development was classified into 11 distinct stages (Figure 3A): undifferentiated spermatogonia (Undiff SPG: *UCHL1*, *UTF1*, and *GFRA1*), early-differentiating spermatogonia (Early-Diff SPG: *KIT*), mid-differentiating spermatogonia (Mid-diff SPG: *PODXL2* and *PCNA*), late-differentiated spermatogonia (Late-diff SPG: *STRA8*), leptotene spermatocytes (Lep SPC: *SMC1B* and *ZCWPW1*), zygotene spermatocytes (Zyg SPC: *SYCP1* and *TEX101*), pachytene spermatocytes (Pach SPC: *PIWIL1* and *MLH3*), diplotene-to-metaphase II spermatocytes (Dip-MII SPC: *CCNB2*, *SPATA16*, and *NME8*), round spermatids (Round SPT: *CREM* and *FHL5*), elongate spermatids (Elongate SPT: *PRM1* and *TNP2*), and immature sperms (Immature SPT: *OAZ3* and *CRISP2*). The cell cycle analysis revealed that the Undiff SPG contained a higher fraction of cells in the G1 phase, while most Dip-MII SPC cells were in the G2/M phase (Figure 3B). *TOP2A*, a cell proliferative marker gene [32], showed strong expression increases in cells from Early-Diff SPG to Pach SPC (Figure 3C). 

### 3.5. Re-Clustering of Shaziling Pig Spermatogonial Cells 

**Figure 4:**
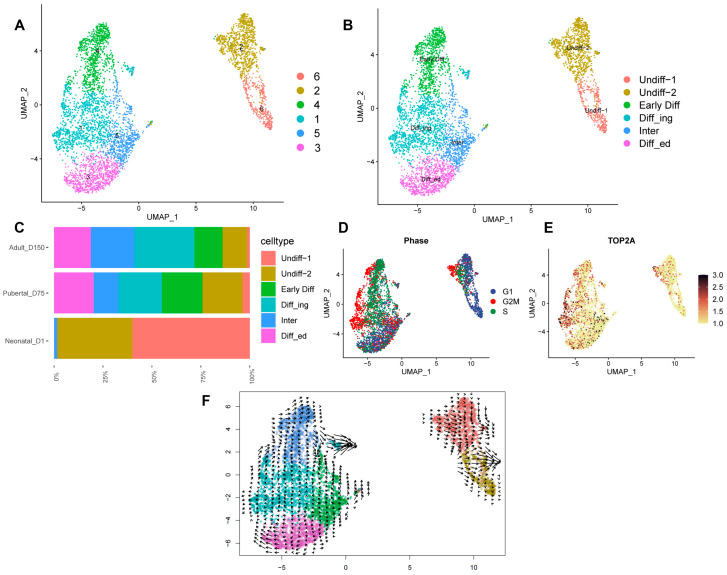
Heterogeneity of spermatogonial cells. The Shaziling pig spermatogonial cells were re-clustered and classified into six distinct cell types. The cell types are labeled with (**A**) corresponding cluster numbers and (**B**) annotated cell-type names: undifferentiated-1 spermatogonia (cluster 6: Undiff-1 SPG), undifferentiated-2 spermatogonia (cluster 2: Undiff-2 SPG), early-differentiating spermatogonia (cluster 4: Early-Diff SPG), differentiating spermatogonia (cluster 1: Diff_ing SPG), intermediate spermatogonia (cluster 5: Inter SPG), and differentiated spermatogonia (cluster 3: Diff_ed SPG). The analyzed spermatogonia were classified into six distinct cell types. The relative proportions of these cell types in D1, D75, and D150 are presented in (**C**). (**D**) Cell-cycle phase analysis of spermatogonial cells. The different phases of the cell cycle, including G1, G2M, and S, are represented by dots of different colors. (**E**) *TOP2A* expression demonstrates spermatogonial cell proliferation. Changes in *TOP2A* gene expression levels from high to low are indicated by a transition in color from dark red to light yellow on the right side of the UMAP. (**F**) Velocity field projected onto the UMAP embedding. The arrows indicate the predicted root states and terminals of the spermatogonia subpopulations.

Re-clustering of male germ cells revealed the presence of multiple developmental stages in SPG; therefore, we extracted and re-clustered SPG to further reveal its heterogeneity. We defined six cell clusters of SPG using cell marker genes, including Undiff-1 SPG (cluster 6: *ZBTB16*, *UTF1*, and *FGFR3*), Undiff-2 SPG (cluster 2: *GFRA1*, *UCHL1*, and *PLD6*), Early-Diff SPG (cluster 4: *KIT*), Diff_ing SPG (cluster 1: *TOP2A* and *PODXL2*), Inter SPG (cluster 5: *KIT*, *TOP2A*, *PODXL2*, and *PCNA*), and Diff_ed SPG (cluster 3: *STRA8*) (Figure 4A,B and Figure S6).

Both Undiff-1 and Undiff-2 subsets exhibited the expression of a set of SSC marker genes, but certain genes, such as *UCHL1* and *GFRA1*, were highly expressed in Undiff-2. Neonatal_D1 samples had the highest proportions of Undiff-1 (59.87%) and Undiff-2 (38.46%), whereas the proportions of Undiff-1 and Undiff-2 decreased sharply to 3.89% and 20.41% in pubertal_D75 and 1.38% and 12.48% in adult_D150 samples, respectively (Figure 4C). 

Cell cycle analysis showed that cells in the Undiff-1 subset were predominantly in the G1 phase, with only a few cells in the synthetic (S) or G2/mitotic (M) phase. In contrast, in Undiff-2, the number of cells in the S phase or G2/M phase was relatively high. This indicated that most of the cells in Undiff-1 were not proliferating, while the cells in Undiff-2 were undergoing slow proliferation (Figure 4D). In addition, *TOP2A* had the lowest expression in the Undiff-1 SPG, whereas its expression increased from the Early-Diff SPG to the Diff_ed SPG (Figure 4E). RNA velocity analysis inferred the existence of two differentiated cell fates for spermatogonia (Figure 4F).

### 3.6. Potential SSC Marker Genes and Important Pathways for Maintenance and Self-Renewal of SSCs 

**Figure 5:**
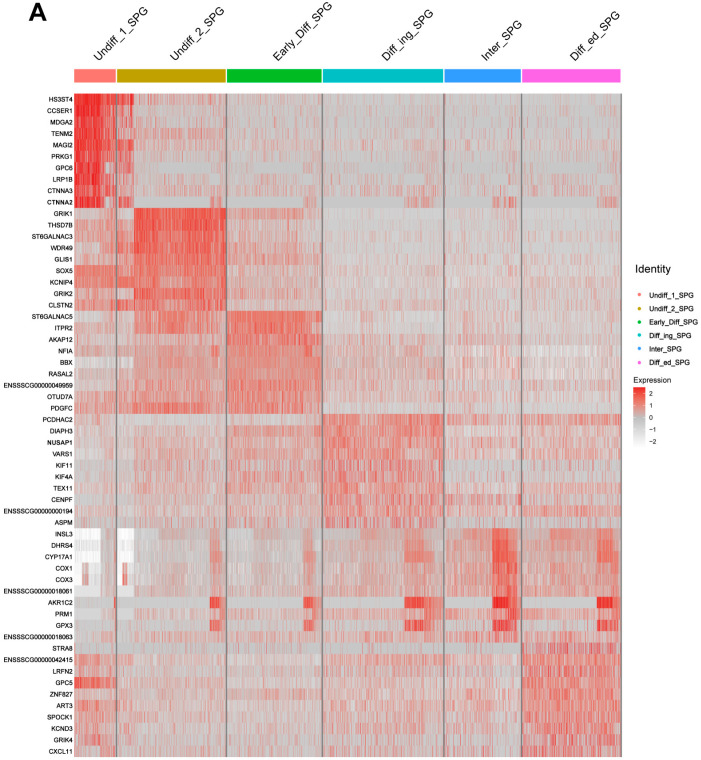

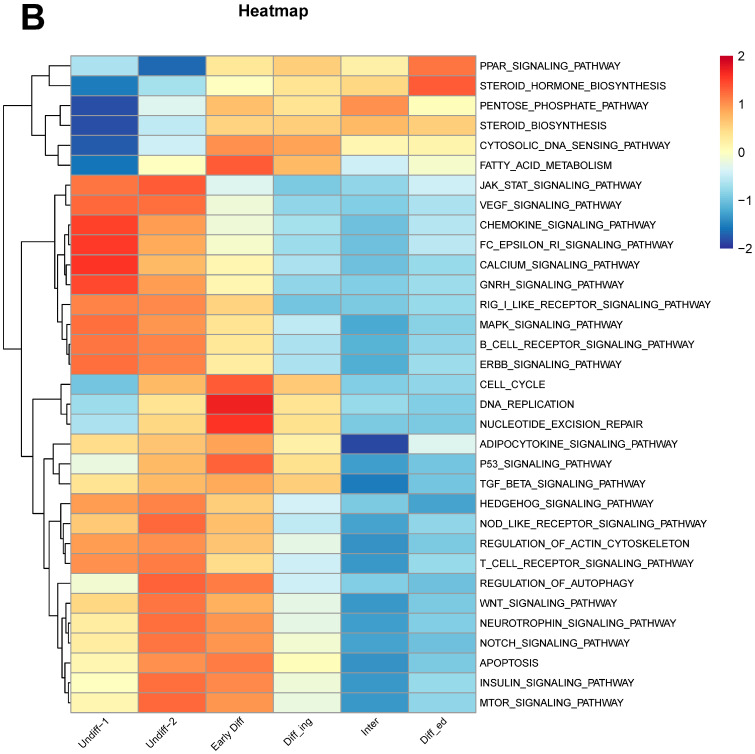
Potential SSC marker genes and important pathways for maintenance and self-renewal of SSCs. (**A**) Heatmap of the top 10 marker genes for each cell type of spermatogonial cells. Different cell types are marked by the different colors at the top of the heat map. The left side of the heatmap displays the gene IDs. Color changes from red to light grey on the right side of the heatmap indicate changes in gene expression levels from high to low. (**B**) The GSVA pathway enrichment analysis indicated several significant up-regulated cell signaling pathways in both Undiff-1 and Undiff-2 SPG subsets. Color changes from dark red to dark blue on the right side of the heatmap indicate the up-regulation and down-regulation of pathways, respectively.

The majority of cells in the Undiff-1 and Undiff-2 SPG subsets expressed mammalian SSC marker genes that had been previously identified in humans [33], mice [34], and pigs [20]. This suggested that these two subsets were enriched for spermatogonial stem cells (SSCs), with Undiff-1 representing a non-proliferative state, while Undiff-2 represented slowly proliferating SSCs. We further generated the gene expression correlation heatmap of spermatogonia to reveal the potential SSC marker genes of Shaziling pigs (Figure 5A). In Undiff-1, the *HS3ST4*, *CCSER1*, *MDGA2*, *TENM2*, *MAGI2*, *PRKG1*, *GPC6*, *LRP1B*, *CTNNA3*, and *CTNNA2* genes were specifically highly expressed, which may serve as the potential SSC functional markers for Shaziling pigs (Supplementary Figure S7). Detailed information on marker genes of the SPG subsets is listed in Supplementary Table S4.

The gene set variation analysis (GSVA)-based pathway enrichment analysis (Figure 5B) indicated several significant up-regulated cell signaling pathways in both Undiff-1 and Undiff-2 SPG subsets, including Jak-Stat, VEGF, chemokine, Fc epsilon RI, calcium, GnRH, MAPK, and ErbB signaling pathways. These up-regulated signaling pathways may be critical for the maintenance and self-renewal of SSCs in Shaziling pigs.

### 3.7. Pseudotime Trajectory Analysis of Shaziling Pig Spermatogonial Cells 

**Figure 6:**
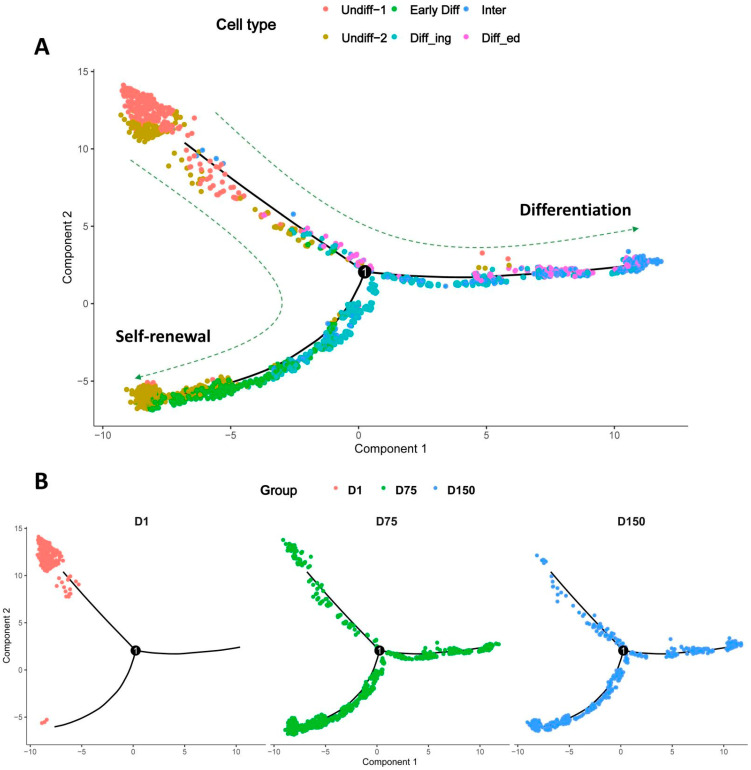

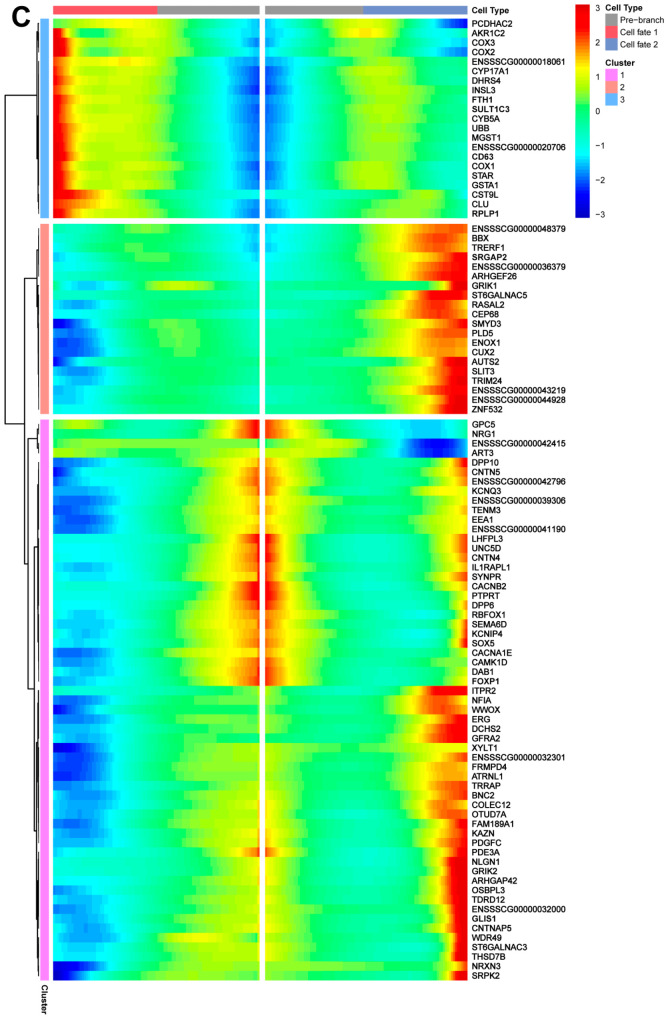
Pseudotime trajectory analysis of spermatogonial cells. (**A**) Pseudotime trajectory analysis reveals two spermatogonial cell developmental trajectories, one representing SSC self-renewal and the other representing SSC differentiation. (**B**) Faceted plots of pseudotime trajectories at D1, D75, and D150. (**C**) BEAM demonstrates cell fate-determining genes associated with spermatogonial cell development.

We utilized Monocle pseudotime trajectory analysis to examine the developmental relationship between the six SPG subsets. By organizing SPG cell subsets along their respective developmental trajectories in accordance with differentially expressed genes, Monocle analysis revealed the existence of two spermatogonial cell developmental trajectories, one representing SSC self-renewal and the other representing SSC differentiation (Figure 6A). In D1 samples, spermatogonia were only in the self-renewal state, whereas in D75 and D150 samples, spermatogonia had already differentiated to accomplish subsequent spermatogenesis (Figure 6B). We performed branch expression analysis modeling (BEAM) to obtain cell fate-determining genes associated with spermatogonial cell development. The dynamic expression fluctuations of these genes are illustrated on the heatmap (Figure 6C). The top five genes that may be associated with maintaining SSC stemness and self-renewal are *NLGN1*, *GRIK2*, *GLIS1*, *PLD5*, and *GFRA2*, and the top five genes that may be involved in driving SSC differentiation are *AKR1C2*, *COX2*, *COX3*, *INSL3*, and *CST9L*. Pseudotime analysis of gene expression showed dynamic changes in spermatogonial cells. 

### 3.8. Developmental Pattern of Spermatogonia in Shaziling Pigs

**Figure 7:**
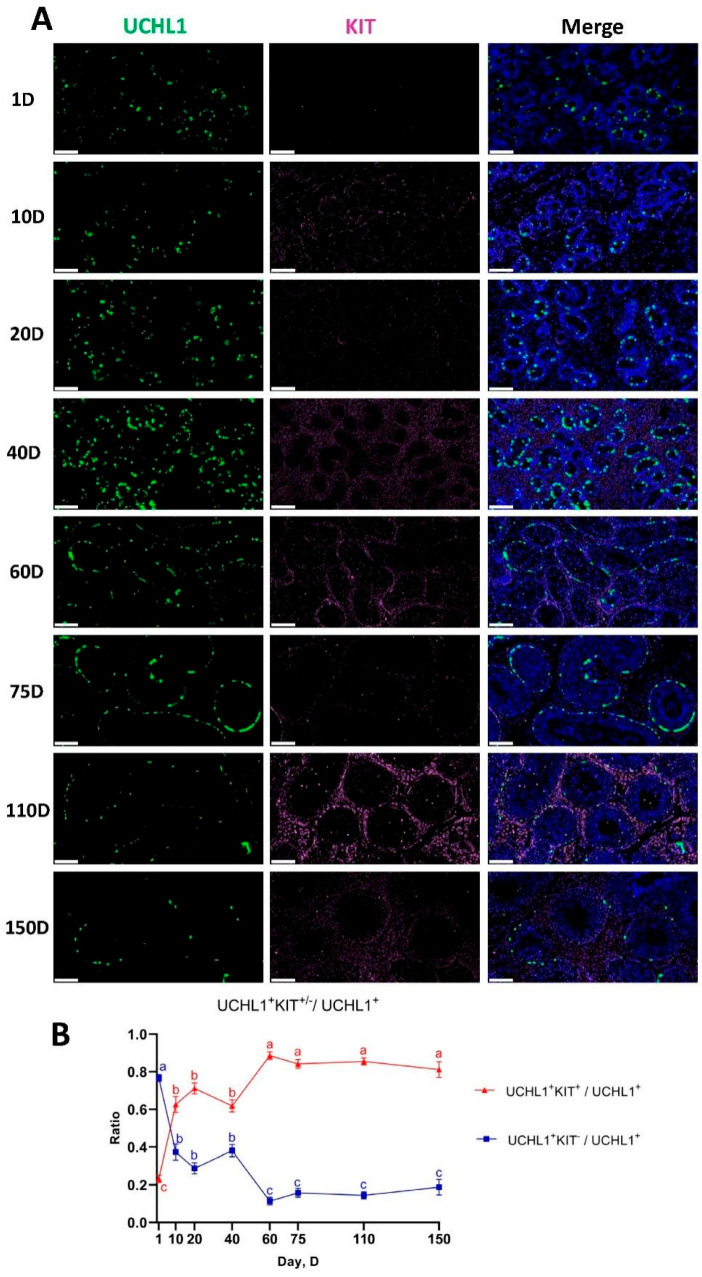
Immunostaining and quantification of UCHL1^+^ and KIT^+/−^ cells in Shaziling pig testis sections. (**A**) UCHL1 and KIT immunostaining of Shaziling pig testis sections at each age. Bar = 100 μm. (**B**) The ratio of UCHL1^+^ KIT^+/−^ to UCHL1^+^ cells in seminiferous tubule cross-sections from Shaziling pigs of eight ages. Data are shown as mean ± SEM of three replicates, with 30 seminiferous tubule cross-sections analyzed per individual. Different letters indicate significant differences between age groups (*p* < 0.05).

To determine the time point of spermatogonial differentiation in Shaziling pig testes, we performed double immunostaining for UCHL1 and KIT on testicular sections from eight different ages (Figure 7A). UCHL1 is a conserved pan-spermatogonial cell marker in testes, and KIT is a pan-marker for differentiating spermatogonia. UCHL1 can serve as a molecular marker for most undifferentiated spermatogonia in mammals [33,35,36], and its expression in pigs is restricted to type A (A_single_, A_paired_, and A_aligned_) undifferentiated spermatogonia [37]. KIT is a transmembrane protein receptor associated with germ cell maturation [38] and a marker of loss of potential in spermatogonial stem cells [39]. It is expressed in differentiated spermatogonia of porcine testis tissues [40] and can be used to identify differentiated spermatogonia of goats [41].

Quantification of UCHL1^+^ and KIT^−^ cells in each section of seminiferous tubules revealed that the average ratio of UCHL1^+^ KIT^−^ cells (which corresponded to the undifferentiated spermatogonia that are enriched for SSCs) to UCHL1^+^ cells was highest (ratio = 0.77) on day 1 (D1) and exhibited a significant decline beginning on D10. Conversely, the average ratio of UCHL1^+^ KIT^+^ (which indicated differentiating spermatogonia) to UCHL1^+^ cells was minimal (ratio = 0.23) on D1 and increased significantly beginning on D10. The double immunostaining results indicated that the spermatogonia of the Shaziling pig differentiated early, from D10, and the ratio of undifferentiated and differentiating spermatogonia remained relatively stable from D60 to D150 (Figure 7B). 

## 4. Discussion

We performed single-cell transcriptomics and histomorphologic analysis of Shaziling pig testes at three key developmental stages. In male mammals, significant alterations in testicular weight and physiology correspond to testicular development. These changes include the differentiation of spermatogonia, the maturation of seminiferous tubules under the support of niche cells, and the regulation of intercellular hormonal signaling, all of which contribute to the establishment of a continuous and stable mechanism for spermatogenesis [1,42]. 

In Shaziling pigs, we resolved the testicular heterogeneity by characterizing the dynamic transcriptome profiles of various cell types during postnatal testicular maturation. The testis is a crucial male reproductive gland responsible for the continuous production of sperm, and it primarily comprises the seminiferous tubules and testicular interstitium. The seminiferous tubules consist of Sertoli cells in the inner part, forming the epithelial layer. Undifferentiated SPGs, which contain SSCs, are found on the basal lamina of the seminiferous tubules. During testicular development, SSCs begin to proliferate, and some SSCs differentiate into differentiated SPGs and initiate spermatogenesis, while others establish a dependable SSC pool [43]. The testicular interstitium contains various types of cells, including Leydig cells, peritubular myoid cells, smooth muscle cells, endothelial cells, and immune cells. Those cells release paracrine and endocrine substances that support the normal development of sperm and maintain direct communications with each other. The interactions between SSCs and various cell types, along with the secretion of multiple cytokines by these cells, establish a microenvironment that supports continuous spermatogenesis [4].

Spermatogonia in boars have been classified into four categories: undifferentiated A spermatogonia (A_single_, A_paired_, and A_aligned_), differentiating A spermatogonia (A_1_, A_2_, A_3_, and A_4_), intermediate spermatogonia, and B spermatogonia [44]. Our single-cell analysis identified six spermatogonial subsets that reflected the heterogeneity of SSCs: Undiff-1 SPG (might correspond to undifferentiated A type: A_single_), Undiff-2 SPG (undifferentiated A type: A_paired_ or A_aligned_), Early-Diff SPG (differentiating A type), Diff_ing SPG (more advanced differentiating A type), Inter SPG (intermediate type), and Diff_ed SPG (B type). Undiff-1 characterized SSCs that did not proliferate, whereas Undiff-2 represented SSCs that proliferated slowly and were subject to self-renewal. The differentiation fate of SSCs was denoted sequentially as Early-Diff SPG, Diff_ing SPG, Inter SPG, and Diff_ed SPG. The intricate balance between self-renewal and differentiation of SSCs is critical for the long-term spermatogenesis process [45]. 

The lack of unique cell markers for porcine SSCs makes the isolation of these cells inefficient and hinders further research. We found several possible novel SSC marker genes (e.g., *MDGA2*, *TENM2*, *MAGI2*, *PRKG1*, *GPC6*, *LRP1B*, and *CTNNA2*) in Shaziling pigs, which may be suitable for serving as cell membrane surface protein markers to isolate SSCs. Although universal marker genes for SSCs exist in mammals [46] and some SSC marker genes have been identified in other pig breeds [20,21,47,48], we proposed the hypothesis that SSC markers may be breed-specific due to the different genetic backgrounds of pig breeds. Several SSC-specific marker genes of Shaziling pigs were identified through further examination of the genes that were specifically highly expressed in the Undiff-1 SPG. For example, *MDGA2* (MAM domain containing glycosylphosphatidylinositol anchor 2) is a class of cell adhesion molecules that have been reported to regulate axon growth [49,50]. *TENM2* (teneurin transmembrane protein 2) is involved in cell adhesion and intercellular signaling [51]. *PRKG1* (protein kinase cGMP-dependent 1) is a key mediator in the cGMP signaling pathway and is involved in many cellular signaling processes [52]. *GPC6* (glypican 6) is involved in the regulation of cell growth and cell division [53,54]. *LRP1B*, which is a member of the low-density lipoprotein (LDL) receptor family, is involved in numerous cellular processes [55,56]. *CTNNA2*, also known as catenin alpha 2, facilitates the binding of actin filaments and is involved in the regulation of neuron migration and the development of neuron projections [57]. It is worth noting that these SSC candidate marker genes have not been experimentally validated in testicular tissues or SSC cells, and the exact functions of these candidate genes in spermatogenesis are not known; so, their reliability as SSC marker genes needs to be further investigated.

The maintenance of the SSC pool is crucial for lifelong spermatogenesis, requiring a delicate balance between self-renewal and differentiation [43]. In particular, the self-renewal and maintenance of SSCs are tightly controlled by external signals in a special niche microenvironment within the seminiferous tubules. We found that the Undiff-1 and Undiff-2 SPG subsets represented SSCs in a state of self-renewal and maintenance in which several signaling pathways were specifically up-regulated. The Jak-Stat signaling pathway is conserved among multiple species and plays a crucial role in regulating various biological processes, including the self-renewal of stem cells [58,59]. Male germline stem cells of Drosophila are maintained by the local activation of the Jak-STAT pathway; alternatively, these stem cells differentiate but fail to self-renew in the absence of Jak-STAT signaling [60]. Early studies suggested that VEGF signaling is involved in promoting angiogenesis [61,62]. Further research has demonstrated its significant role in spermatogenesis and the maintenance of SSCs [63,64]. The inhibition of chemokine signaling in the testes of adult mice results in the depletion of SSCs, which are essential for the maintenance of the SSC pool in mice [65]. Calcium signaling is crucial for spermatogenesis and sperm motility [66,67]. Moreover, the disruption of calcium signaling has a significant association with male infertility [68]. However, the functional role of calcium signaling in SSCs is yet to be determined. Studies have shown that the inhibition of GnRH promotes the colonization and proliferation of transplanted SSCs in mice [69], rats [70], and monkeys [71,72]. 

Using pseudotime trajectory analysis, we also found several candidate genes that were associated with maintaining SSC stemness and self-renewal. Notably, our list of candidate genes includes many genes associated with brain-related traits. For example, *NLGN1*, which stands for neuroligin 1, is a cell surface protein that functions in synaptic signal transmission and facilitates cell–cell interactions through its interactions with members of the neurexin family [73,74]. A glutamate receptor *GRIK2* gene is the excitatory neurotransmitter receptor most commonly found in the mammalian brain. Its activation is involved in numerous regular neurophysiologic processes [75,76,77]. *GLIS1*, also known as GLIS family zinc finger 1, is a Kruppel-like zinc finger protein that serves dual roles as a transcription activator and repressor [78]. *PLD5* encodes a member of the phospholipase D family, which is an isoform of the transphosphatidylase enzyme and is linked to diverse cellular effects, including cell growth, survival, and migration [79,80]. *GFRA2*, which stands for neurturin receptor alpha 2, is a crucial regulator of neuron survival and differentiation [81,82]. In addition to being highly expressed in human brain tissues, these above-mentioned genes are also highly expressed in testicular tissues [83]. However, until recently, the role of these genes in maintaining the stemness and self-renewal of SSCs remained unknown. Our study suggests that there is a great need to investigate the function of these genes in SSCs.

## 5. Conclusions

We employed snRNA-Seq analyses to investigate heterogeneity in testicular development from the newborn stage to maturity in Shaziling pigs. Using over 80 thousand testicular cells, a total of 10 distinct cell types were defined, consisting of germ cells, somatic cells, and immune cells. Several putative marker genes were screened for each cell type. Furthermore, our study focused on the developmental patterns of spermatogonia. During intact spermatogenesis, we elucidated the complex dynamic processes governing SSC development within their niche in the Shaziling pig testes. We identified several potential genes and cellular signaling pathways associated with the regulation processes of SSC self-renewal and maintenance. We also suggested several potential marker genes for SSCs that could be used for subsequent isolation and sorting for SSCs from Shaziling pigs. Our investigation resulted in the generation of an invaluable genetic resource that enhanced our understanding of spermatogenesis and the development of testes in Shaziling pigs after birth.

## Data Availability

All datasets presented in this study are included in the article/Supplementary Material. The sequencing data have been uploaded to the National Genomics Data Center database under Bioproject No. PRJCA023782 (https://ngdc.cncb.ac.cn/bioproject/ accessed on 18 April 2024).

## Supplementary Materials

The following supporting information can be downloaded at: https://www.mdpi.com/article/10.3390/biom14060607/s1, Figure S1: Histological examination of testicular tissue sections of D1, D75, and D150; Figure S2: Immunofluorescence staining showing UCHL1^+^ cells in porcine testicular sections at three stages of testicular developmen; Figure S3: The individual UMAP plots; Figure S4: The UMAP plots of cell marker genes for the major cell types; Figure S5: The GO BP enrichment analysis identified the functional categories associated with each annotated cell type; Figure S6: The individual UMAP plots of each replicate after re-clustering of Shaziling pig spermatogonia cell; Figure S7: The UpSet plot demonstrated the gene set characteristics of SPG subsets; Table S1: The snRNA-seq data summary; Table S2: The relative abundance of each cell type in the three developmental stages of the Shaziling pig testis samples; Table S3: Detailed cell type-specific marker gene information; Table S4: Detailed information about marker genes for the SPG subsets.
