# Supplementary material for: Single-Nucleus RNA-Seq Reveals Spermatogonial Stem Cell Developmental Pattern in Shaziling Pigs
Source: Biomolecules. 2024 May 21;14(6):607. doi: 10.3390/biom14060607 (PMC11202124; doi:10.3390/biom14060607)
Supplement: Supplementary file 1 [file biomolecules-14-00607-s001.zip › 20240519 Tang et al Supplementary Info.pdf]

Supplementary Information

Single-nucleus RNA-Seq reveals spermatogonial stem cell development pattern of Shaziling pig

Xiangwei Tang [1], Chujie Chen [1], Saina Yan [2], Anqi Yang [1, 3], Yanhong Deng [1], Bin Chen [1 *], and Jingjing Gu [1 *]

[1] College of Animal Science and Technology, Hunan Provincial Key Laboratory for Genetic Improvement of Domestic Animal, Hunan Agricultural University, Changsha 410128, China.

[2] College of Animal Science and Technology, China Agricultural University, Beijing 100193, China.

[3] School of Basic Medical Sciences, Hengyang Medical School, University of South China, Hengyang 421001, China.

* Correspondence author:

E-mail addresses: chenbin7586@hunau.edu.cn (B. Chen), jingjing.gu@hunau.edu.cn (J. Gu).

Supplementary Figures

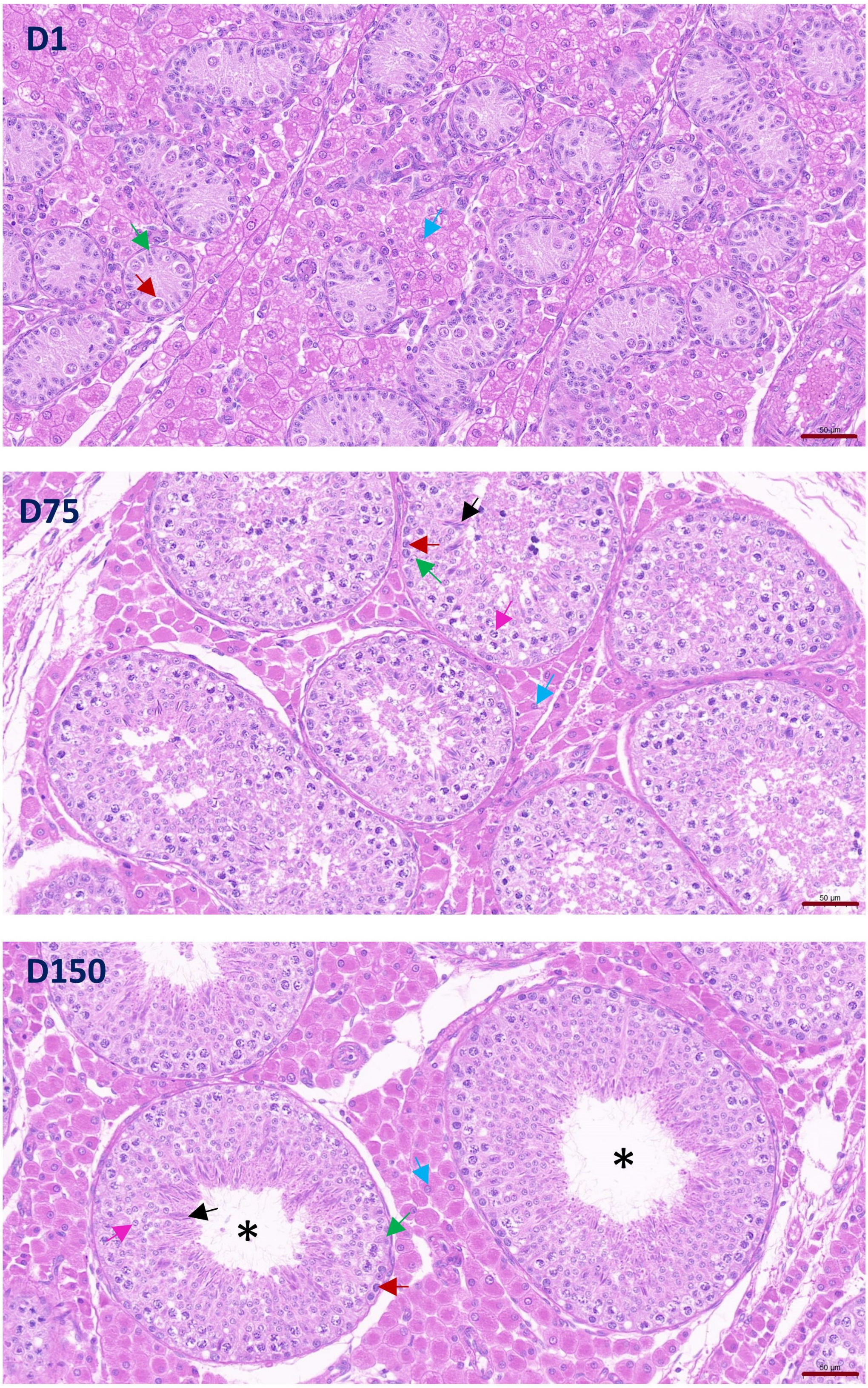

Supplementary Figure S1. Histological examination of testicular tissue sections of D1, D75, and D150. Blue arrow, Leydig cells; green arrow, Sertoli cells; red arrow, spermatogonia; pink arrow, spermatocytes; black arrow, spermatids; *, lumens of seminiferous tubules. Bar = 50 μm.

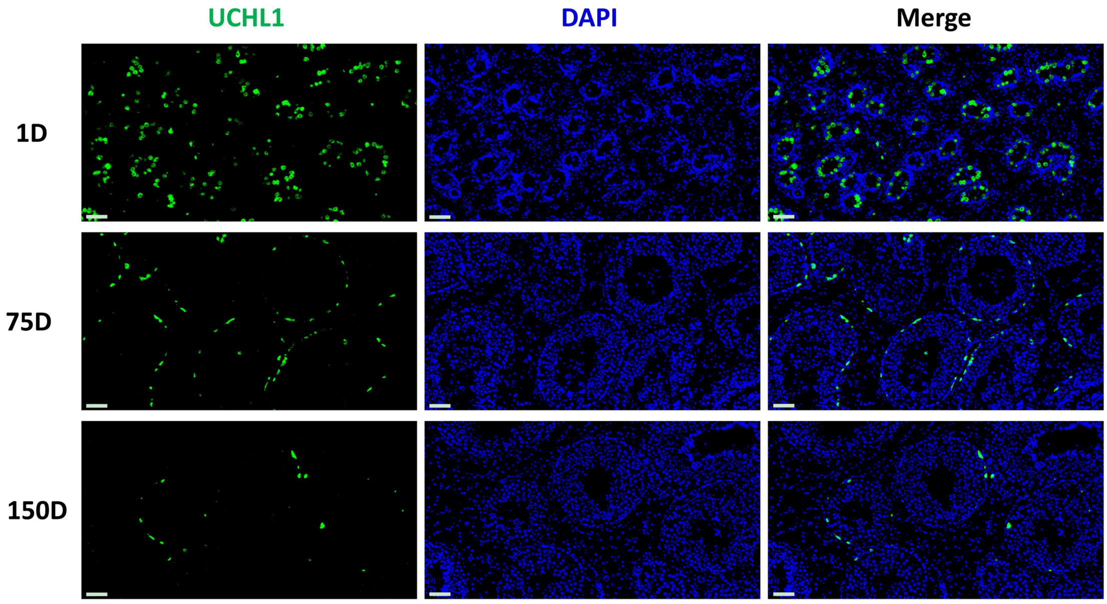

Supplementary Figure S2. Immunofluorescence staining showing UCHL1^{+} cells in porcine testicular sections at three stages of testicular development. Bar = 100 μm.

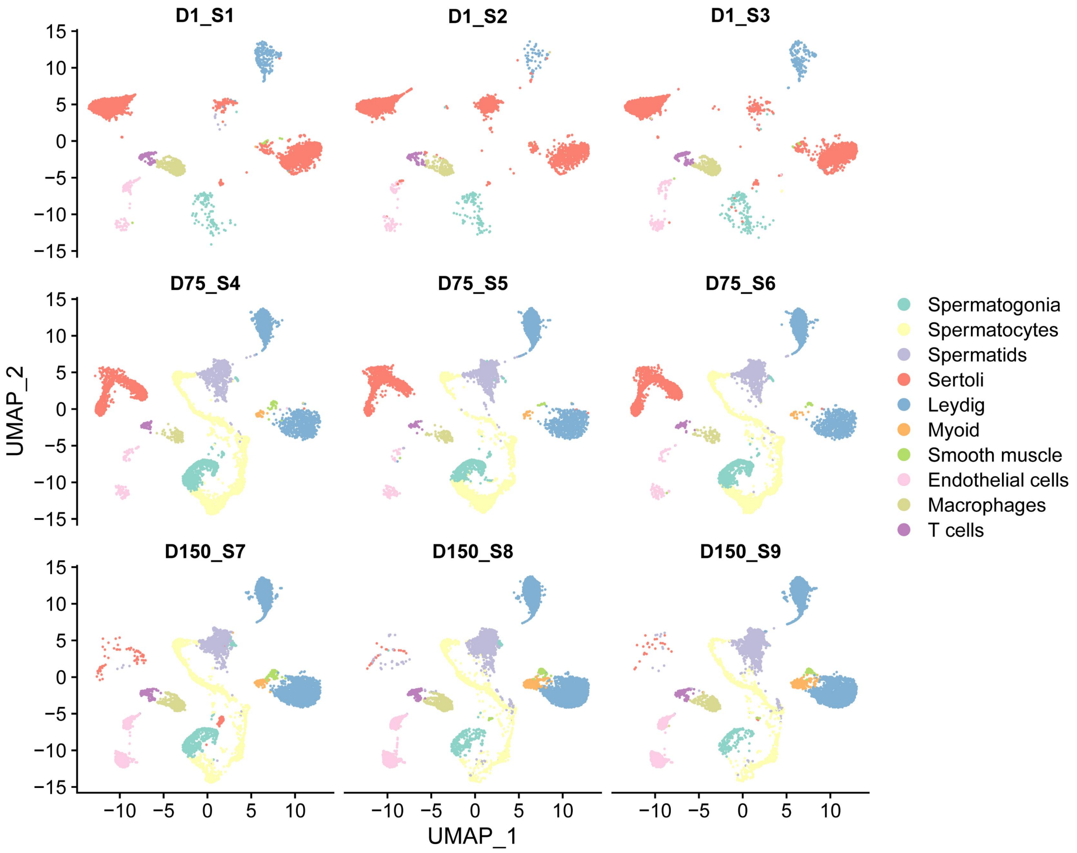

Supplementary Figure S3. The individual UMAP plots of each replicate.

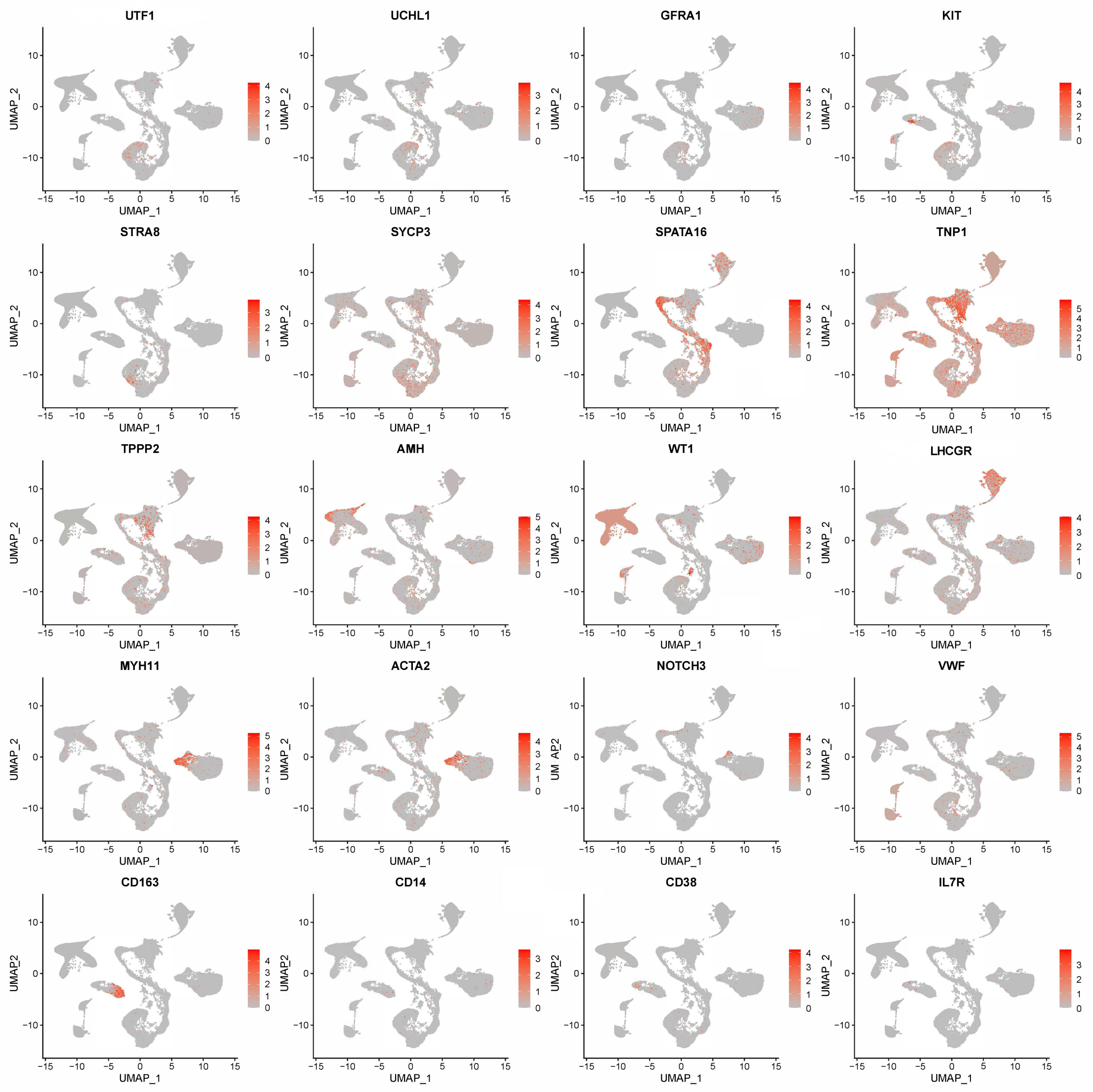

Supplementary Figure S4. The UMAP plots of cell marker genes for the major cell types.

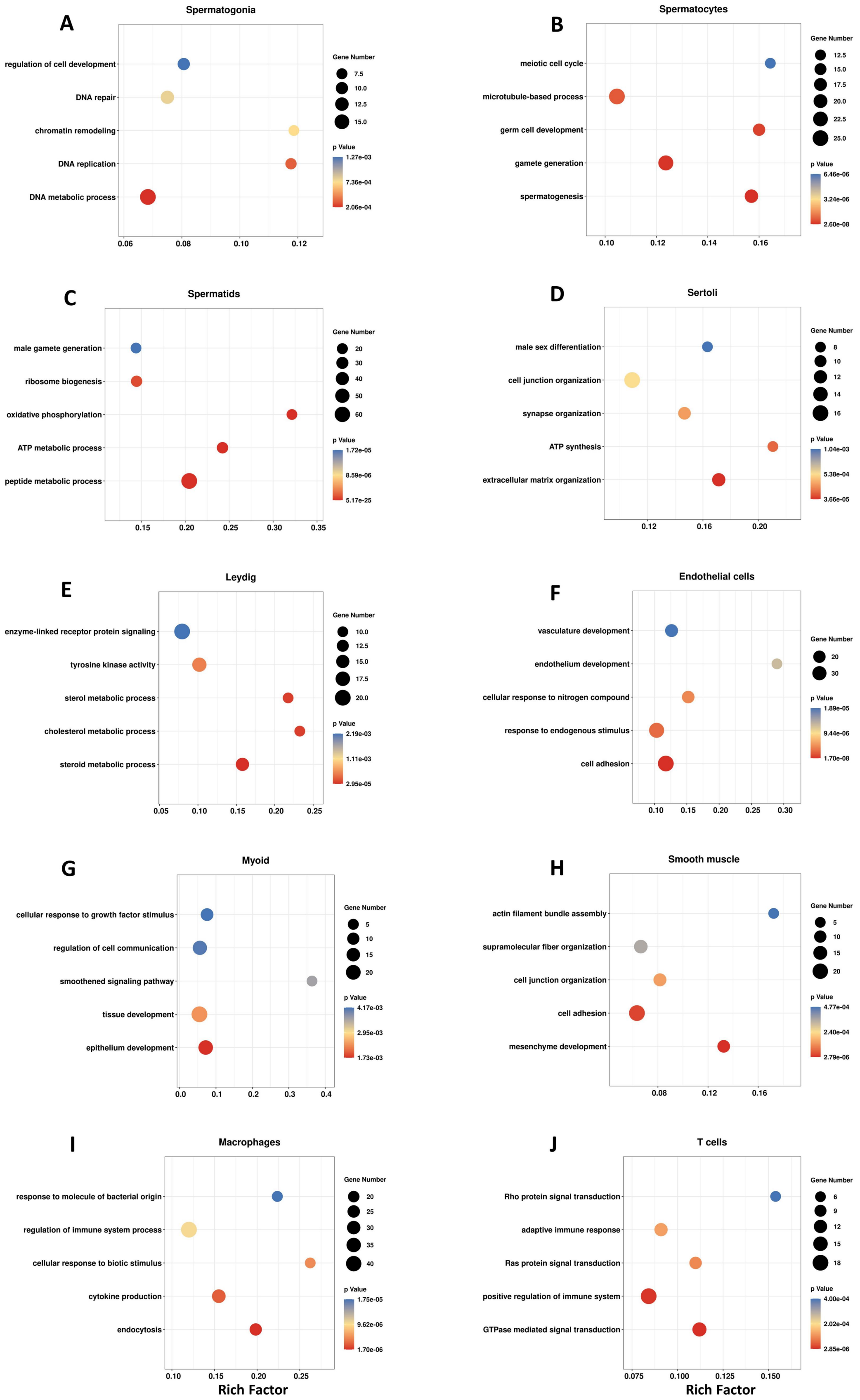

Supplementary Figure S5. The GO BP enrichment analysis identified the functional categories associated with each annotated cell type.

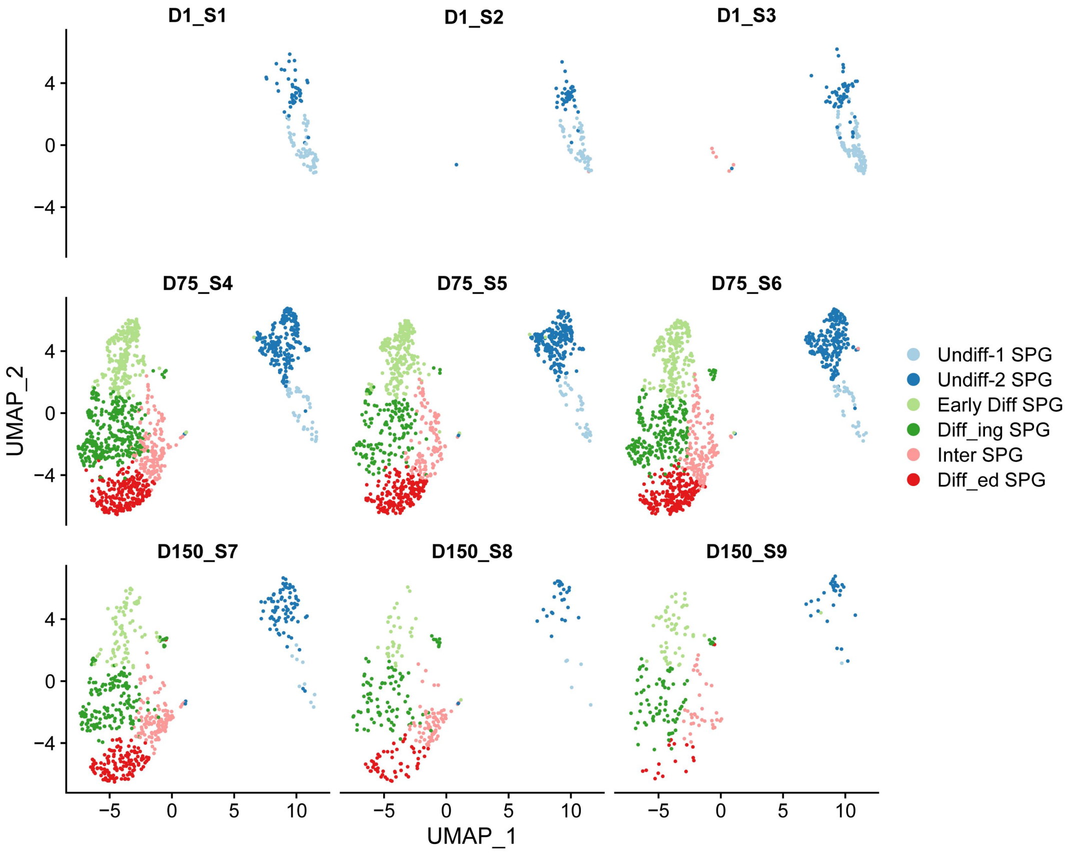

Supplementary Figure S6. The individual UMAP plots of each replicate after re-clustering of Shaziling pig spermatogonia cells.

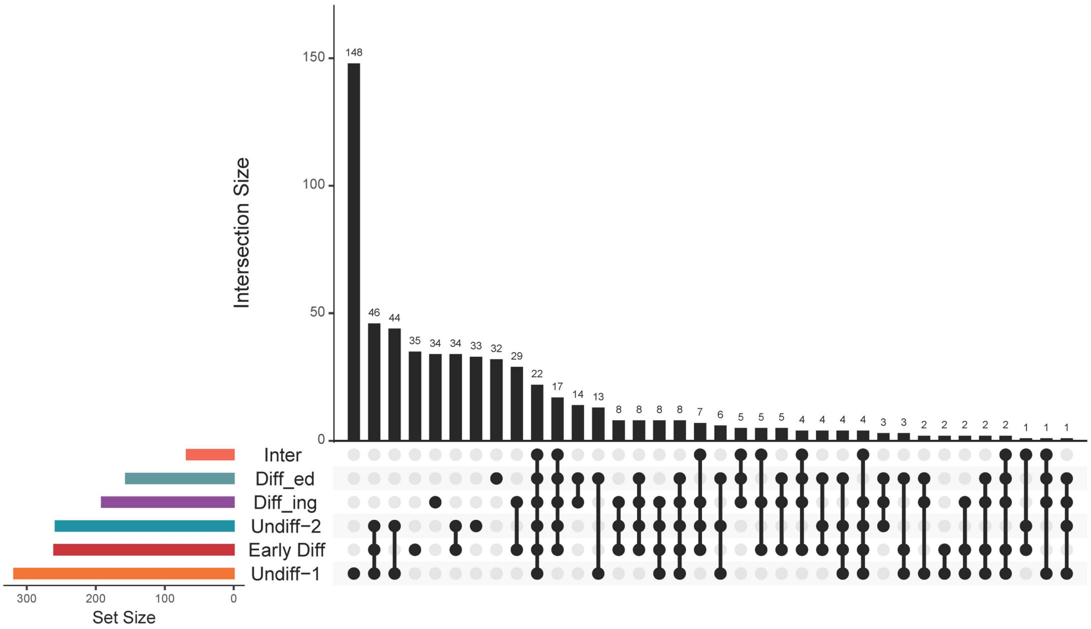

Supplementary Figure S7. The UpSet plot demonstrated the gene set characteristics of SPG subsets.

Supplementary Tables 1-2

Supplementary Table S1. The snRNA-seq data summary.

Estimated Total Number of Cells	82,171
Mean Reads per Cell	39,584
Median Genes per Cell	1,918
Number of Reads per Sample	349,545,339
Valid Barcodes	97.74%
Sequencing Saturation	58.09%
Q30 Bases in Barcode	95.72%
Q30 Bases in RNA Read	94.00%
Q30 Bases in UMI	94.84%
Reads Mapped to Genome	92.32%
Reads Mapped Confidently to Genome	88.53%
Reads Mapped Confidently to Intergenic Regions	14.53%
Reads Mapped Confidently to Intronic Regions	45.83%
Reads Mapped Confidently to Exonic Regions	28.16%
Reads Mapped Confidently to Transcriptome	52.00%
Reads Mapped Antisense to Gene	20.68%
Fraction Reads in Cells	54.87%
Total Genes Detected	26,805
Median UMI Counts per Cell	3,118

Supplementary Table S2. The relative abundance of each cell type in the three developmental stages of the Shaziling pig testis samples.

	**Developmental stage**		
**Cell type**	**Neonatal_D1**	**Pubertal_D75**	**Adult_D150**
Spermatogonia	2.91%	13.95%	4.12%
Spermatocytes	not found	31.67%	15.29%
Spermatids	not found	10.68%	17.96%
Sertoli	84.22%	20.00%	1.46%
Leydig	3.28%	19.66%	38.11%
Myoid and smooth muscle	0.16%	0.73%	4.86%
Endothelial cells	1.32%	1.21%	11.95%
Immune cells (macrophages and T cells)	8.11%	2.10%	6.25%
